# Optimization of Lipase Production by *Burkholderia* sp. Using Response Surface Methodology

**DOI:** 10.3390/ijms131114889

**Published:** 2012-11-13

**Authors:** Chia-Feng Lo, Chi-Yang Yu, I-Ching Kuan, Shiow-Ling Lee

**Affiliations:** Department of Bioengineering, Tatung University, No 40, Sec. 3, Zhongshan N. Rd., Taipei 104, Taiwan; E-Mails: alexnws@hotmail.com (C.-F.L.); chrisyu@ttu.edu.tw (C.-Y.Y.); iching@ttu.edu.tw (I.-C.K.)

**Keywords:** lipase, *Burkholderia* sp., optimization, response surface methodology

## Abstract

Response surface methodology (RSM) was employed to optimize the extracellular lipase production by *Burkholderia* sp. HL-10. Preliminary tests showed that olive oil, tryptone and Tween-80 exhibited significant effects on the lipase production. The optimum concentrations of these three components were determined using a faced-centered central composite design (FCCCD). The analysis of variance revealed that the established model was significant (*p* < 0.01). The optimized medium containing 0.65% olive oil (*v*/*v*), 2.42% tryptone (*w*/*v*) and 0.15% Tween-80 (*v*/*v*) resulted in a maximum activity of 122.3 U/mL, about three fold higher than that in basal medium. Approximately 99% of validity of the predicted value was achieved.

## 1. Introduction

Lipases (triacyl glycerol acylhydrolases, EC 3.1.1.3), catalyzing the hydrolysis of acylglycerides to fatty acids, di-acylglycerides, mono-acylglycerides and glycerols [1], have received considerable attention recently. They have been extensively used in food, cosmetic, textile, medical and industrial applications owing to their versatile catalytic activities of triglycerides hydrolysis, esters synthesis, trans-esterification and resolution of racemic mixtures [1–3]. Lipases were found to be produced by many varieties of organisms. However, lipases from microbial sources are of great importance due to their diverse commercial applications both in aqueous and non-aqueous systems [4].

Medium composition plays a crucial role in the lipase production [5]. A general practice to maximize the lipase activity is by varying one factor at a time while keeping others at constant levels. Nevertheless, this method does not depict the interactive effects among the factors and it does not locate the optimum conditions of processes. Experimental statistical techniques such as response surface methodology (RSM), however, provide an effective alternative to the conventional approach in many biotechnological processes [6–9]. RSM can reveal the correlations between the factors and responses as well as the optimum level of each factor employed [10]. Optimization of lipase production by RSM has been reported in cultures of *Cadida* sp. 99–125, *Pseudomonas aeruginosa* and *Bacillus pumillus*[11–13].

*Burkholderia* sp. HL-10 used for this study was isolated from lipid-contaminated soils [14]. *Burkholderia* sp., well-known for production of alkaline lipases, has been widely applied in the biodegradation of environmental pollutants [15]. Lo in his preliminary study found that medium components exhibited marked effects on lipase production by *Burkholderia* sp. HL-10 [14]. Herein, a central composite design of response surface approach was used to examine the interaction among medium components including olive oil, tryptone and Tween-80, and to determine their optimum concentrations in order to yield a maximum lipase activity.

## 2. Results and Discussion

### 2.1. Central Composite Design and Response Surface Analysis

Preliminary screening of medium composition indicated that olive oil, tryptone and Tween-80 exhibited significant effects on lipase production by *Burkholderia* sp. HL-10. One-factor-at-a-time approach was used to identify the concentration levels of these three parameters [14]. Most lipases are inducible enzymes and addition of oils proved to enhance lipase production [13,16]. Olive oil was the most effective compared to soybean oil, palm oil, canola oil and sunflower oil [14]. Besides, as a nitrogen source, tryptone is rich in minerals and ions which has also been reported to improve the lipase activity [17]. Tween-80 not only acted as an effective surfactant but also a lipase inducer [13]. Because RSM can serve as a successive and exploratory tool for establishing the interaction of variables [16,18], a set of 20 experiments designed with the faced-centered central composite design (FCCCD) were carried out to determine their optimum concentrations for maximum lipase production. The design matrix of the variables in coded units and actual concentrations along with the experimental response (lipase production) is presented in Table 1.

The results obtained were then subject to the analysis of variance (ANOVA) to establish a response surface quadratic model. As formulated in Equation 1, lipase activity (*Y*) was expressed as a function of concentrations of olive oil (*X*_1_), tryptone (*X*_2_) and Tween-80 (*X*_3_).

(1) $$\begin{array}{c} Y=102.71+6.83\;X_{1}+33.33\;X_{2}+1.95\;X_{3}+9.50\;X_{1}X_{2}-0.87\;X_{1}X_{3}\\ -0.60\;X_{2}X_{3}-12.02\;{X_{1}}^{2}-21.01\;{X_{2}}^{2}+1.33\;{X_{2}}^{3} \end{array}$$

As shown in Table 2, the model generated was statistically significant with a value of “Probability > *F*” less than 0.05, suggesting the lipase activity could be well described with this model. The lack of fit measures the unfitness of the model to represent data within experimental region. Therefore, the non-significant lack-of-fit (*p* = 0.5978) indicated the model was significant [18] and the equation was suitable for simulation of lipase production with any combination of three variables. The value of *R^2^* was 0.9866 revealing a relatively high correlation between experimental and predicted values and 98.66% of the variability in the response could be explained by the model [19]. The predicted *R^2^* of 0.9091 was in accordance with the adjusted *R^2^* of 0.9746, also supporting that the regression model could be used to describe the response trends [20]. The adequate precision which measured the ratio of signal-to-noise was 26.244, higher than 4, again confirming the model adequacy [19–21]. Thus, this model could be used to navigate the design space satisfactorily.

**Figure 1:**
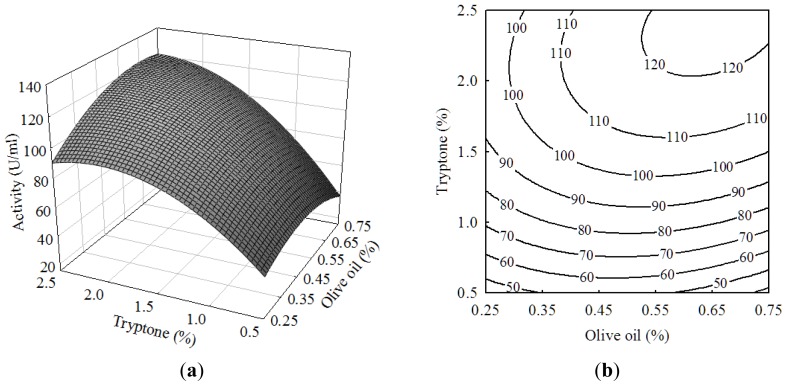
(**a**) Response surface curve and (**b**) Contour plot showing the effects of olive oil and tryptone on the lipase activity of *Burkholderia* sp. HL-10.

**Figure 2:**
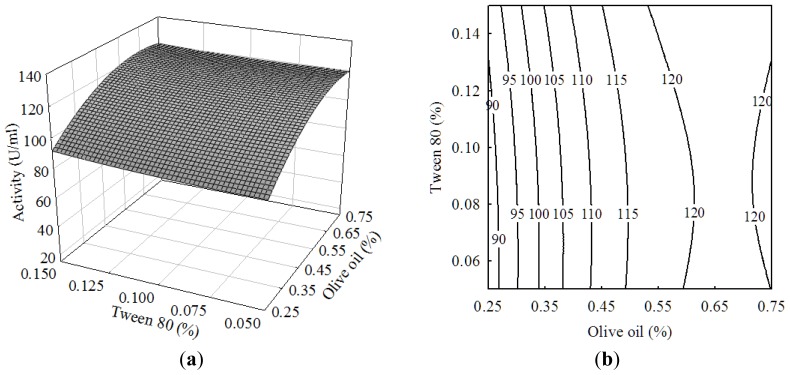
(**a**) Response surface curve and (**b**) contour plot showing the effects of olive oil and Tween-80 on the lipase activity of *Burkholderia* sp. HL-10.

**Figure 3:**
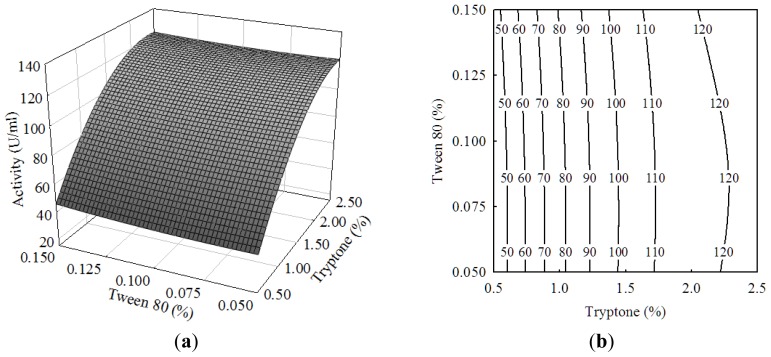
(**a**) Response surface curve and (**b**) contour plot showing the effect of tryptone and Tween-80 on the lipase activity of *Burkholderia* sp. HL-10.

The three-dimensional response surface curves and contour plots in Figures 1–3 showed the interactions amongst olive oil, tryptone and Tween-80. Two variables were investigated at a time while keeping the other one at a fixed concentration. Figure 1 showed a somewhat elliptical contour, suggesting a synergistic effect between olive oil and tryptone. In the range studied, higher concentrations of olive oil and tryptone markedly enhanced the production of lipase. This was consistent with the fact that lipases were generally induced in presence of oils [21,22], and tryptone was reported to enhance the lipase activity of *Acinetobacter calcoaceticus*[17] and *Yarrowia lipolytica*[23].

Tween-80 as a surfactant can lower the interfacial tension between oils and water and increase cell permeability, thus possibly facilitating the enzyme secretion [20]. Figure 2 showed the correlation between olive oil and Tween-80. Unexpectedly, an increase in Tween-80 concentration did not exert an obvious effect on lipase activity. It might be attributed to the lower concentration of Tween-80 of 0.05% to 0.15%. In the previous reports on the lipase production by *Bacillus pumilus*[13], *Bacillus sp.*[24] and *Rhizopus oligosporous*[25], Tween-80 concentration ranging from 0.5% to 2% was generally added in the medium. Similar results were observed with the interactive effect between tryptone and Tween-80 (Figure 3).

The optimum concentrations for olive oil, tryptone and Tween-80 as obtained from the maximum point of the polynomial model were 0.65% (*v*/*v*), 2.42% (*w*/*v*) and 0.15% (*v*/*v*), respectively. In turn, the maximum activity predicted was 122.8 U/mL.

### 2.2. Verification of Model

The response surface model was validated with additional experiments under the predicted conditions. The experimental value obtained was 122.3 U/mL (the average of triplicates), which was very close to the predicted value of 122.8 U/mL. Approximately 99% of validity was achieved, indicating the model exerted an adequate prediction on the enzyme activity.

### 2.3. Lipase Production in Basal and Optimized Media

**Figure 4:**
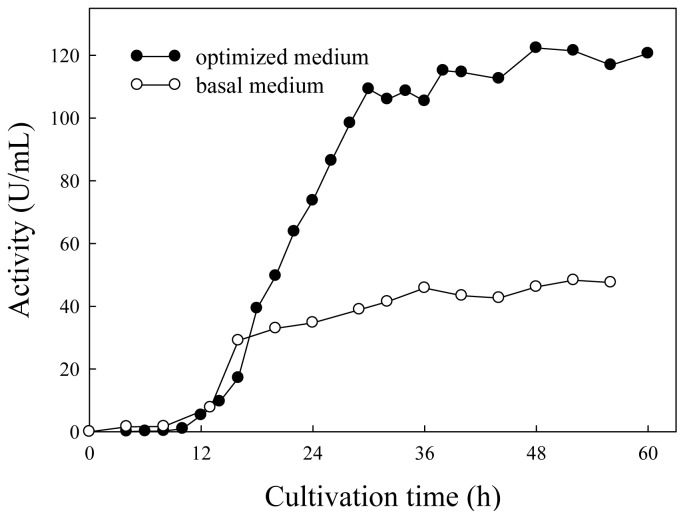
Lipase production by *Burkholderia* sp. in basal and optimized media.

Figure 4 showed the time course profile of the lipase activity for *Burkholderia* sp. HL-10 cultivated in basal and optimized media. The lipase production started at late log phase of the bacterial growth in both media. The maximum lipase activity of 45.8 U/mL in basal medium was observed after 36-h cultivation, while the activity maximum of 122.3 U/mL in optimized medium was achieved at 48 h. Optimization of culture media, therefore, led to an increase in lipase yield by about three fold. The results obtained in this study were in accordance with those from previous related studies. Dandavate *et al.*[16] showed that the lipase activity of *B. multivorans* V2 was enhanced by almost 2.2 fold after optimization of the culture medium using RSM. Liu *et al.*[26] reported similar optimization with RSM for *Burkholderia* sp., which resulted in a 5-fold increase in lipase production. RSM was also performed for *Bacillus pumilus*[13] and *Rhizopus delemar*[20], giving rise to an increase in lipase production by 3.14 and 3.25 fold, respectively.

## 3. Experimental Section

### 3.1. Bacterial Strain and Preparation of Inoculum

The bacteria strain used in this study was isolated from lipid-contaminated soil samples collected in Tatung University. Identification was carried out by morphological and biochemical methods and identified as *Burkholderia* sp. [14]. The organism was grown on Luria-Bertani (LB) agar slant which consisted of 1% tryptone (*w*/*w*), 1% NaCl (*w*/*w*), 0.5% yeast extract (*w*/*w*) and 1.5% agar (*w*/*w*). The slants were incubated at 30 °C for 24 h. The inoculum was prepared by aseptically transferring a loop full of cells from the agar slant into 50 mL LB medium. The cultivation was then carried out at 30 °C on an orbital shaker at 200 rpm for 10 h to yield a microbial density of *ca*.10^9^ CFU/mL.

### 3.2. Culture Conditions for Lipase Production

Two hundred microliters of the inoculum prepared above was added to 50 mL medium in a 250 mL Erlenmeyer flask. The basal medium for lipase production consisted of 1% (*v*/*v*) soybean oil, 1% (*w*/*v*) tryptone, 0.1% (*v*/*v*) Tween 80, 0.02% (*w*/*v*) yeast extract, 0.02% (*w*/*v*) NaNO_3_ and 0.02% (*w*/*v*) MgSO_4_ with a pH of 6.5 adjusted by 1 M HCl [15]. According to the results obtained from one-factor-at-a-time approach, the medium employed in the design matrix of FCCCD was composed of 0.5% olive oil, 1.5% (*w*/*v*) tryptone, 0.1% (*v*/*v*) Tween-80, 0.06% (*w*/*v*) yeast extract, 0.02% (*w*/*v*) NH_4_CH_3_COO and 0.05% (*w*/*v*) MgSO_4_[14]. The culture was incubated at 30 °C with orbital shaking at 200 rpm for 48 h. The lipase activity in the culture supernatant was then measured after centrifugation at 12,500*g* for 10 min at 4 °C.

### 3.3. Response Surface Methodology

The statistical approach using FCCCD developed by the Design Expert software (Version 6.0.1, Stat-Ease Inc., Minneapolis, USA) was used to generate a set of 20 experimental runs with six replicated center points. Three different levels, low (−1), medium (0) and high (+1) were used to study the independent variables (olive oil, tryptone and Tween-80), and the lipase activity was taken as a dependent variable (*Y*) (Table 1). A quadratic polynomial regression model was established to describe the relationship between dependent and independent variables:

(2) $$Y=\beta_{0}+\beta_{1}\;X_{1}+\beta_{2}\;X_{2}+\beta_{3}\;X_{3}+\beta_{12}\;X_{1}\;X_{2}+\beta_{13}\;X_{1}\;X_{3}+\beta_{23}\;X_{2}\;X_{3}+\beta_{11}\;{X_{1}}^{2}+\beta_{22}\;{X_{2}}^{2}+\beta_{33}\;{X_{3}}^{2}$$

where *Y* is the predicted response (lipase activity); *X*_1_, *X*_2_ and *X*_3_ are the independent variables (olive oil, tryptone and Tween-80 concentrations); β_0_ is the intercept, β_0_, β_1_ and β_3_ are the linear coefficient; β_12_, β_13_ and β_23_ are the interaction coefficient; β_11_, β_22_ and β_33_ are the quadratic coefficients. Analysis of variance (ANOVA) was performed to evaluate the adequacy of the model. Three-dimensional response surface curves were plotted by Design Expert software to display the interaction among various variables.

### 3.4. Assay for Lipase Activity

Lipase activity was monitored using *p*-nitrophenyl palmitate (*p*-NPP) as substrate according to the method described by Pencreac’h and Baratti [27] with minor modification. The assay mixture was prepared by adding 90 μL of 8.25 mM *p*-NPP in isopropanol and 810 μL of solution containing 50 mM Tris-HCl buffer (pH 8.0) with 0.494% Triton X-100 and 0.12% arabic gum, followed by pre-incubation at 40 °C for 5 min. The enzymatic reaction was then initiated by adding 100 μL of appropriate diluted lipase sample (culture supernatant) and proceeded at 40 °C for 5 min. The amount of *p*-nitrophenol released was measured at 410 nm using a Helios-α spectrophotometer equipped with a thermostatic cell holder (Unicam, Cambridge, UK). One unit (U) of lipase activity is defined as the amount of enzyme that liberates 1 μmol *p*-nitrophenol per minute at 40 °C. All experiments were performed in triplicate.

## 4. Conclusions

The present work aimed to optimize lipase production by *Burkholderia* sp. HL-10 with response surface methodology. The *R^2^* value of 0.9866 for the model established clearly indicated a relatively high correlation between model and experimental data. The optimum concentrations of olive oil, tryptone and Tween-80 were determined to be 0.65% (*v*/*v*), 2.42% (*w*/*v*) and 0.15% (*v*/*v*), respectively, which gave rise to almost a three-fold increase in the maximum lipase activity. Thus, this work demonstrated the feasibility of statistical methodology to develop optimum medium composition with a minimum number of experimental trials.

## Figures and Tables

**Table 1:** Face-centered central composite design of the medium components in coded and actual units for the lipase production.

Run	*X*_1_ (olive oil)		*X*_2_ (tryptone)		*X*_3_ (Tween-80)		Activity (U/mL)
		
	coded	actual (%)	coded	actual (%)	coded	actual (%)	
1	−1	0.25	−1	0.5	−1	0.05	39.3
2	1	0.75	−1	0.5	−1	0.05	31.3
3	−1	0.25	1	2.5	−1	0.05	81.7
4	1	0.75	1	2.5	−1	0.05	120.8
5	−1	0.25	−1	0.5	1	0.15	42.6
6	1	0.75	−1	0.5	1	0.15	40.2
7	−1	0.25	1	2.5	1	0.15	91.6
8	1	0.75	1	2.5	1	0.15	118.3
9	−1	0.25	0	1.5	0	0.10	85.5
10	1	0.75	0	1.5	0	0.10	98.3
11	0	0.50	−1	0.5	0	0.10	45.8
12	0	0.50	1	2.5	0	0.10	120.0
13	0	0.50	0	1.5	−1	0.05	105.2
14	0	0.50	0	1.5	1	0.15	105.2
15	0	0.50	0	1.5	0	0.10	97.2
16	0	0.50	0	1.5	0	0.10	107.6
17	0	0.50	0	1.5	0	0.10	98.3
18	0	0.50	0	1.5	0	0.10	98.2
19	0	0.50	0	1.5	0	0.10	101.8
20	0	0.50	0	1.5	0	0.10	108.5

**Table 2:** Analysis of variance (ANOVA) for the quadratic model of lipase activity of *Burkholderia* sp.

Source	Sum of Squares	DF	Mean square	*F* Value	Probability > *F*
Model	1,6535.33	9	1837.26	82.01	<0.0001
Residual	224.03	10	22.40	20.82	
Lack of fit	99.02	5	19.80	0.79	0.5978
Pure error	125.01	5	25.0		
Corrected total	1,6759.36	19			

*R*^2^ = 0.9866, *R*^2^_pred_ = 0.9091, *R*^2^_adj_ = 0.9746, adequate precision = 26.244
